# Early diastolic leftward septal motion in Tetrology of Fallot at MRI: analysis and comparison to patients with constrictive/restrictive physiology

**DOI:** 10.1186/1532-429X-11-S1-P66

**Published:** 2009-01-28

**Authors:** Jeremy D Collins, Karen Ordovas, Allison Meadows, Maureen Kohi, Brett Elicker, WR Webb, Charlie Higgins

**Affiliations:** UCSF, San Francisco, CA USA

**Keywords:** Constrictive Pericarditis, Short Axis Image, Restrictive Cardiomyopathy, Pulmonary Insufficiency, Regurgitant Fraction

## Background/purpose

Early diastolic leftward septal motion (EDSM) is a nonspecific finding at diagnostic cardiac imaging. Although touted as a sensitive marker for constrictive or restrictive physiology, EDSM can also be seen in volume overload and rhythm disturbances. The purpose of this study was to retrospectively evaluate early leftward diastolic septal motion in patients with Tetrology of Fallot (TOF) and right ventricular enlargement.

## Methods

The UCSF Committee on Human Research approved this study. Medical records and cardiac MR images from 116 consecutive patients with suspected constrictive/restrictive physiology (64 pts, mean age 49 yrs, 34 men), history of surgical repair of TOF with pulmonary insufficiency (PI) (31 pts, mean age 27 yrs, 18 men) or both PI and stenosis (PS) (21 pts, mean age 32 yrs, 10 men) referred for cardiac MRI between January 1997 and September 2008 were reviewed. Images from 9 volunteers (4 men, mean age 29 yrs) were also included in the analysis.

Cinegraphic cardiac MRI images were reviewed for the presence of EDSM, defined as > 2 mm leftward deviation on two consecutive short axis images immediately following isovolumetric relaxation. Ventricular chamber volumes were obtained from contoured short axis images and adjusted to body surface area. The extent of pulmonary insufficiency was assessed with velocity encoding sequences orthogonal to the right ventricular outflow tract. Images were reviewed for pericardial thickening, enhancement, effusions, and coupling of the parietal and visceral pericardial layers as well as evidence for impaired diastolic filling. QRS complex duration, pulmonary valve gradient, and systemic venous and pulmonary wedge pressures were obtained from the medical record, when available. An analysis of variance (ANOVA) was performed on TOF patients with p > 0.05 considered statistically significant. Results from pathology, right heart catheterization, echocardiography and surgical procedures provided the reference standard for analysis.

## Results

EDSM was identified in 58% (18 of 31) TOF patients with PI and 48% (10 of 21) patients with PI and PS. Grouping all TOF patients with PI for ANOVA, right ventricular end diastolic volume index was strongly associated with EDSM (p = 0.001); QRS complex duration (p = 0.18), pulmonary regurgitant fraction (p = 0.61), and left ventricular end diastolic volume index (LVEDVI) (p = 0.32) demonstrated weaker associations. Right atrial and pulmonary wedge pressures were available in 16 TOF patients, precluding evaluation with ANOVA. See Table 1.

**Table 1:** Table 1

	TOF Patients c EDSM	
RV EDVI	PI only	PI and PS
Normal	0% (0 of 4)	0% (0 of 2)
Mildly Enlarged	63% (5 of 8)	30% (3 of 10)
Moderately Enlarged	63% (7 of 11)	66% (4 of 6)
Severely Enlarged	75% (6 of 8)	100% (3 of 3)

EDSM was identified in 18 of 21 patients (86%) with suspected (5 patients) or proven (16 patients) constrictive pericarditis and in 6 of 12 patients (50%) with suspected (3 patients) or proven restrictive cardiomyopathy (9 patients). Eight individuals out of 39 (30 patients, 9 volunteers) without MR or corroborative evidence of constrictive pericarditis or myocardial pathology demonstrated EDSM; 7 patients were subsequently diagnosed with a volume overload state.

## Conclusion

A substantial proportion of patients with TOF and enlarged right ventricular volumes demonstrated EDSM at CINE cardiac MRI, controlling for effects from QRS complex duration, LVEDVI and pulmonary regurgitation. This finding, although somewhat less prevalent in our TOF sample population than in constrictive pericarditis, suggests that progressive enlargement of the right ventricle may lead to an appearance mimicking constrictive physiology. We hypothesize that in TOF patients, development of EDSM is due to reduced compliance of the stretched pericardium. Careful prospective assessment of volume status in TOF patients will be necessary to validate these findings and enable correlation of EDSM with development of clinical symptoms.

